# Through Measurement to Knowledge: The Inaugural Lecture of Heike Kamerlingh Onnes (1882)

**DOI:** 10.6028/jres.107.021

**Published:** 2002-06-01

**Authors:** Arno Laesecke

**Affiliations:** National Institute of Standards and Technology, 325 Broadway, Boulder, CO 80305-3328 U.S.A.

**Keywords:** history, metrology, simulation, standards, theory, thermophysical properties

## Abstract

This paper is a contribution to the NIST Centennial 2001. It presents the first complete English translation of the inaugural speech of Heike Kamerlingh Onnes on the occasion of his appointment as Professor at the University of Leiden (The Netherlands) in 1882. The speech is a snapshot of the scientific landscape of that time and lays out a vision. It advocates with enthusiasm the significance of quantitative measurements and the development of metrology standards. Although science and technology have advanced since then by orders of magnitude, a number of interesting parallels between then and now appear.

## 1. Introduction

Richard P. Feynman opened his visionary lecture “There’s Plenty of Room at the Bottom” in 1959 as follows: “I imagine experimental physicists must often look with envy at men like Kamerlingh Onnes, who discovered a field like low temperature, which seems to be bottomless and in which one can go down and down. Such a man is then a leader and has some temporary monopoly in a scientific adventure.” [1]. Why did Feynman refer to Kamerlingh Onnes first? Perhaps because Kamerlingh Onnes had a vision of similar magnitude as his own. Feynman anticipated, or rather challenged, the development of micro- and nanotechnology which is now—40 years later—coming to fruition and will experience accelerated growth in coming decades. Kamerlingh Onnes laid out his vision in his inaugural lecture on November 11, 1882, titled *De betekenis van het quantitatief onderzoek in de natuurkunde* (The Significance of Quantitative Investigations in Physics), on the occasion of being appointed professor of experimental physics at the University of Leiden (The Netherlands).

This contribution presents the first complete English translation of Kamerlingh Onnes’ inaugural lecture, in which he coined the famous phrase *door meten tot weten* (Through measurement to knowledge). Many may ask “Why bother 120 years after the event?” The author believes that the history of scientific achievements is important. As James Bryant Conant, research chemist and president of Harvard University from 1933 until 1953, wrote, “To understand science requires that one retraces the steps by which certain end results have been produced, not that one simply be exposed to a logical exposition of the subject. That is, it requires a historical approach.” [2]. This is the purpose of the present and many previous historical accounts of scientific developments, among which J. S. Rowlinson’s retracings of van der Waals’ works are particularly close to the present account [3, 4]. With a similar objective in mind, Ottino [5] compared aspects of creativity in the arts and science. He notes that in science the foremost interest is placed on the “final picture and not the perspiration.” Unlike in the arts, the genesis of an idea and its evolution is rarely appreciated in science. But just as a great painting does not happen in a flash, scientific progress is achieved not only by the proverbial “flash of mind”.^1^ It evolves also. Ironically, modern information technology is prone to reduce the historical context and the evolution of scientific achievements. If employed without consideration of its incomplete coverage of the past, information technology gives the user an illusion of being informed, rather than all the information s/he needs [6]. As a result, those uninformed about the past run the risk of reinventing the wheel. Furthermore, “being well informed about science is not the same thing as understanding science.” [2].

The evolution of Kamerlingh Onnes’ work can be traced over a longer period of time than that of most other researchers. Publication of the *Communications from the Physical Laboratory at the University of Leiden* began already in 1885, only 3 years after his appointment as professor. There is, however, still an earlier reference of Kamerlingh Onnes that marks the beginning of the evolution of his work more appropriately: the inaugural lecture of 1882 that he delivered when he took the office of professor of experimental physics. Kamerlingh Onnes presented in this visionary lecture his agenda, the program that he would execute in the following decades. It was printed, but only in Dutch, and was thus of limited availability for the historic research on Kamerlingh Onnes.

An English translation of this lecture appears useful for three reasons. First, it provides the missing piece to appreciate fully the evolution of Kamerlingh Onnes’ achievements. Second, the lecture is a snapshot of an era when metrology was being established as a scientific discipline in its own right. It is timely to recall these developments on the occasion of the Centennial of the National Institute of Standards and Technology, the national metrology institute of the United States of America, which is rooted in that era. Finally, a historical account provides not only contemplation of the past but orientation for the future as well.

In the following, brief biographical information about Kamerlingh Onnes and his early career until 1882 is contained in Sec. 2. His inaugural lecture is put into perspective with a sketch of contemporary cultural developments during that era. These introductory remarks are followed by the central part of this paper, the first complete English translation of Kamerlingh Onnes’ inaugural lecture of 1882. Although expressed 120 years ago, several key statements of the lecture sound surprisingly current. An interpretive discussion is offered in an epilogue.

## 2. Biographical and Historical Notes

So that the reader may appreciate the significance of Kamerlingh Onnes’ inaugural lecture, it is useful to provide biographical information concerning the most essential milestones of his career until 1882. Excellent accounts of his life have been published by Cohen [7], de Bruyn Ouboter [8] and Mendelssohn [9]. The last one concentrates on Kamerlingh Onnes’ scientific career from 1882 until the first liquefaction of helium in 1908 and the award of the Nobel Prize in 1913. It is particularly insightful because it describes not only scientific achievements but reveals also the personalities and “perspirations” of the principal investigators in the field of low-temperature physics, among whom Kamerlingh Onnes was truly unique. His inaugural lecture is briefly quoted in the short biography by van den Handel [10]. The most widely available and easily accessible account of Kamerlingh Onnes’ life is the concise summary at the web site of the Nobel Foundation [11].

Heike Kamerlingh Onnes was born as the first of three siblings on September 21, 1853, at Groningen, The Netherlands. His birth order has not been mentioned in earlier biographies except in a personal remark quoted by Cohen [7]. It is worthwhile to pay attention to this aspect in view of Sulloway’s finding of a significant correlation between the birth order of scientists and their scientific achievements [12]. Kamerlingh Onnes was educated first at the *Hoogere Burgerschool* of his birth-place (secondary school without classical languages), and subsequently received instruction in Greek and Latin. In 1870 he entered the University of Groningen, obtained his *candidaats* degree (approx. B.Sc.) the following year, and then went to the University of Heidelberg in Germany until April 1873 to study with Robert Wilhelm Bunsen and Gustav Kirchhoff, who jointly developed the method of spectroscopy. After his return to Groningen, he passed his *doctoraal* examination (approx. M.Sc.) in June 1876 and obtained the doctor’s degree in 1879 with a thesis on *Nieuwe bewijzen voor de aswenteling der aarde* (New proofs for the axial rotation of the earth) based on his work with Kirchhoff. Already in 1878 he became assistant of Johannes Bosscha, the director of the Polytechnic in Delft, for whom he substituted as lecturer in 1881 and 1882. At the remarkably young age of 29 years he was appointed Professor of Experimental Physics at Leiden University in 1882 and became one of the successors of P. L. Rijke. On Saturday, November 11 of that year, he gave his inaugural lecture *De betekenis van het quantitatief onderzoek in de natuurkunde* (The Significance of Quantitative Investigations in Physics). To appreciate the distinction of this appointment and the significance of Leiden as a scientific center, the reader is referred to the very detailed biography by Kipnis et al. [13] of Kamerlingh Onnes’ close friend Johannes Diderik van der Waals.

Kamerlingh Onnes’ choice of this topic was probably motivated by the establishment of rigorous metrology as a scientific discipline during this era. The international metric convention, held from 1870 to 1875, created measurement standards of greater accuracy for length and mass. Seventeen nations took part in this convention. In 1875, the Convention of the Meter was signed; this set up a permanent organization to change the metric system as necessary. This organization, the *Bureau International des Poids et Mésures* (BIPM), is located at Sèvres, near Paris (France). In 1887, the first national metrology laboratory was established in Berlin (Germany) as the *Physikalisch-Technische Reichsanstalt*, and Hermann von Helmholtz accepted, although hesitantly, the position as its first president. The United States was the second of the seventeen original signatories of the Convention of the Meter to establish a national metrology laboratory. The National Bureau of Standards was founded on March 3, 1901 in Washington, DC, with Samuel W. Stratton serving as its first director until 1922.

Of course, this was the age of the Industrial Revolution, with such momentous innovations as the telephone by Alexander Graham Bell in 1876, the incandescent light bulb by Thomas A. Edison in 1879, and the first gasoline-powered vehicles by Gottlieb Daimler and Karl Benz in 1885. Impressionism dominated the visual arts from about 1870 to 1910 and reflected the concurrent industrial developments. One of its main protagonists, Auguste Rodin, completed his brooding sculpture *The Thinker* in 1889. There were equally significant developments in music. For instance, Modest Mussorgsky wrote *Pictures at an Exhibition* in 1874, and Johannes Brahms completed the *Academic Festival Overture* in 1881. The *Adventures of Huckleberry Finn*, generally considered Mark Twain’s greatest work, was published in Great Britain in 1884 and in the United States in 1885. These are some of the developments that surrounded the inaugural lecture of Heike Kamerlingh Onnes in the spirit of making observation material, quantitative. They are quoted here because we share John Prausnitz’ view “that what happens in the culture around us is sure to affect how we practice our profession.” [14]. Thomas S. Kuhn noted earlier that science and technology are social activities and not the domain of solitary, eccentric practitioners. Science cannot be undertaken in isolation, and therefore one cannot disregard its history [15].

The following English translation was elaborated with the combined knowledge of Dutch, English, and German of the translator and of the author, seeking a balance between fidelity to the flavor of the original while making it understandable to present-day readers. Since there is no exact one-to-one correspondence among languages, translation inevitably involves interpretation [16]. However, this was kept to a minimum and no attempt was made to clarify perceived ambiguities in the speech of Kamerlingh Onnes or to use current knowledge to improve on him. Curly brackets { } are used henceforth to indicate additions by the author.

## 3. Translation of *De betekenis van het quantitatief onderzoek in de natuurkunde*

### The Significance of Quantitative Investigations in Physics

#### Speech Delivered at the Accession to the Office of Professorship at the State University at Leiden on Saturday 11 November 1882 by Dr. H. Kamerlingh Onnes Leiden—E. J. Brill {Publisher} 1882

Curators, Professors, Doctors, Students of this University, all, who honor me with your presence,

Very appreciated audience!

The fertility of physics leads to the generation of the means of material well-being and her^2^ predominant influence on our world view leads to the pure spirit of empirical philosophy. She can maintain her important contribution to the thinking and working of the present society only if she continues to wrest new ground from the unknown by observation and experiment.

The number and especially the resources of the institutions that present her with opportunities to that end, are, however, far inferior to her great social importance, to which promotion they are dedicated. He who accepts the important task of educating to the practice of physics and administering one of these institutions has to examine with doubled seriousness his understanding of the requirements for empirical investigations in the present time.

For his work and striving he might know no other stimulus than the poetical thirst for truth; the fathoming of the nature of things might be a life’s aim for him; he can draw the courage to accept an office that presents him the opportunity for those labors only from a conviction of his ability to be useful by maintaining certain principles.

In my opinion the striving for quantitative research, which means the discovery of measurable relationships among the phenomena, must be in the foreground of the experimental study of physics.

*Through measuring to knowing*^3^ is the motto which I would like to write above every physics laboratory.

So pay me your kind attention as I try to elucidate the significance that quantitative investigation has had for the development of physics, and will have in the near future.

Mostly inscrutable are the ways that led the greatest minds to the truth. With an artist’s inspiration they behold her light, they develop questions that entire generations of observers and mathematicians can work to elucidate, and they lay foundations upon which the edifice of science rises. It is not given to everyone to be the greatest architect. But many others are needed to whose labor the care for the solidity of the whole is entrusted. To him whose judgment is sharpened, his hand trained and his character formed, is given the fulfilling privilege to contribute to the completion of grand creations as if by a Newton, and a Huygens, of a Volta, and a Fresnel, of a Faraday or a Kirchhoff. The shape given to every building stone is an expression of their minds.

But for that purpose one must always return to a study of their trains of thought in any experiment when new experience is gained. And then, one feels that their striving for quantitative investigation may not be the only guide for original work, but surely qualitative ideas as well guide us to avoid wasted effort and misconceptions.

Already at the beginning of the scientific era of physics, Galileo set forth the rule, that one has first to measure the phenomena before one can summarize them with an explanatory concept.

His observation of the equal duration of the swings of a pendulum revealed early on the pioneering genius in him. Yet, he did not stop with this accidental observation, but by probing nature with further measurements he succeeded in relating the pendulum’s motion to measure and number {quantitative results} and to deduce the law by means of which he calculated the height of the arch in the dome at Pisa from the period of oscillations of the chandeliers suspended therein. And not only in the phenomena of the pendulum did he discover regularity; the investigation of motion along a sloping plane convinced him after many measurements that all phenomena of gravity could be described in the most simple way by means of uniformly accelerated motion. And thus the basic ideas of inertia and acceleration became the common cornerstones of mechanics and physics.

In the discovery of such laws of nature as those found by Galileo, the significance of quantitative investigations is striking. The mutually related values of two measurable quantities in the same phenomenon, such as time and covered distance in free fall, can constitute a new law by their mutual connection in a simple formula.

Yet the numeric values obtained from measurements can shed light on the phenomena in still another way. By their correspondence with other phenomena that have already acquired a certain significance for us, they can reveal an unexpected connection.

Such a connection was found for instance by Faraday in his investigation of the degree of dissociation of different chemical species by a galvanic current. By correlating the amounts of the dissociated species with their chemical equivalencies after the passage of the same quantity of electricity, he could state his law of specific electrochemical action (according to which with every valence of a chemical atom a specific quantity of electricity, an atom of electricity so to speak, moves through the fluid).

Permit me to give you another example of a third way in which quantitative investigation can contribute to knowledge of the phenomena of nature. When it indicates a steadfast relation between one measurable quantity that disappears and another that appears simultaneously, the supposition will be obvious that the one quantity is converted into the other.

This reasoning was applied in the deduction of the law of conservation of energy. Count Rumford undertook already a large-scale physical experiment in order to demonstrate the conversion of work into heat. The bore of a cannon barrel was enlarged with a mill and the barrel confined in a calorimeter. Although he himself apologizes for the glee he displayed when he saw the water becoming warmer, this apology was certainly not needed when one realizes that he also called attention to the quantity of work that was delivered by the steam engine to the bore and was converted into heat in the center of the gun. Thereby he could estimate the work needed to supply one calorie of heat to be one thousand foot-pounds.

Still, it was proven only by the series of experiments of Joule that a constant relation exists between heat and work in all their conversions. Extremely ingenious measurements of the generation of heat in the galvanic circuit, by combustion, by magneto-electric currents, by compression of gases, and by friction, enabled him to determine this conversion constant between heat and work. It is not surprising that, on the basis of these experiments, he not only enunciated the law of conservation of energy, but also, on the basis of work by Helmholtz, introduced it as a principal law of physics.

The effort of Joule to establish the mechanical equivalent of heat by more and more refined methods is certainly the best evidence of the significance which this man of genius attributed to quantitative investigation. Through his work he earned himself a monument, surely deserving to be honored by the term, Joule’s equivalent.

It must be partly attributed to the absence of such experimental support that the initial earlier contemplations about this law of nature by the profound thinker Mayer were not acknowledged for some time.

But it doubly teaches how gratifying the task of those is, who, renouncing premature contemplations about a new perspective that can be opened by a hypothesis, through perseverance and devotion compare the truth of the hypothesis with experience, and thereby build ever firmer pillars to support the edifice of science.

Only to the extent that we have confirmed our insights by measurement can they become certainty.

Being mindful of *omnia in mensura, in numero et pondere* {everything in measure, number and weight (Lat.)}, the ultimate object of our labor must always be to bring the phenomena into measurable form. This ultimate goal has to govern the entire course of experimental investigation.

In the examples that we considered so far, it was possible to proceed to measurement almost immediately. But often, at first view, it appears as if measurement is out of the question. However, reasoning about a phenomenon should not be abandoned before it is purified, simplified and combined with others in such a way that the interplay of cause and effect becomes apparent in terms of measurable quantities. He who adopts this guiding principle will always direct his attention to the factors that one can change into measurable ones, and he will thereby be led to the right choice of the form of the experiment, a choice which constitutes usually the greatest part of the intellectual effort of an investigation.

This is clearly demonstrated in the history of galvanism.

While Galvani’s name is of only historical interest in physics now, Volta discovered the law of electroscopic differences and unlocked the rich source of Voltaic electricity. Certainly this has to be attributed to the fact that he focused his attention on the measurable change of the voltage difference when different metals are in contact.

We find a similar example in the investigation with which Coulomb paved the way for explanations in the area of electric and magnetic phenomena. He succeeded in finding the measurable element in the oftentimes extremely weak forces that occur in this field. Through accurate investigation of them, he arrived at the knowledge of the fundamental laws of electricity and magnetism. In order to measure these actions he developed the torsion-balance, which has since been applied in every discipline to measure weak forces.

Our present insight in electrical phenomena could, however, be acquired only when the theory of forces inversely proportional to the square of the distance was elaborated by Gauss, Poisson and Green into one of the most beautiful fruits of modern mathematics.

In the course of the mathematical investigation, attention was thus directed to a new measurable quantity, which has become the most widely used in the theory of electrical phenomena.

I mean the electric potential, which for this theory has a significance similar to that of temperature for the theory of heat. Just as a body can be heated to this or that temperature, it can also be charged to a higher or lower potential; and when two points of different potential are connected by a conductor, electricity will flow to that side where the potential is lower, just as heat moves to the place of lowest temperature.

After Thomson dedicated his genius to the improvement of electrostatic measuring instruments, we obtained absolute electrometer apparatuses, from which the potential can be read nearly as sharply and easily as the temperature on a thermometer. The unparalleled suitability, accuracy and sensitivity of these instruments has made the electric potential a tangible quantity. And in order to realize how important the introduction of the potential has been in electric measurements, one needs only to remember how most minds in this area had only a nebulous understanding of electromotive force, voltage and electroscopic difference, and how finally by Thomson’s potentiometers the most delicate investigations became possible. One needs to recall only Boltzmann’s determination of the dielectric permittivity of gases, which Faraday could not discover even with his outstanding perseverance.

Already here we see how theory and experiment mutually fertilize each other, how they support and develop each other. Their cooperation is so profound that it would be futile to ask which of them deserves priority. Sometimes theory took the lead and required experiment only to determine the dimensions that had to be attributed to her ideas. I mention here only the determination of the wavelength of light. At other times, theory relies on the picture that emerges from quantitative investigation.

A remarkable example of such quantitative investigation is that through which Faraday learned to describe the electric and magnetic phenomena in a completely new way instead of by immediate action at a distance.

By an unusual application of the null-method he showed that all electrically charged particles are attracted to charges of the opposite sign, an interaction which can always be represented, as it were, by invisible induction lines. Therefore, the induction might be considered as the fundamental form of electric phenomena. By accurate measurements with the torsion-balance, he managed to follow the induction lines in their curved course through space and could show that the amount of induction depends on the medium with which that space is filled.

Thus he came to the conviction that the medium takes the most important role in the induction, and that the forces that electrically charged bodies exert on each other do not have to be ascribed immediately to an action in distance. Rather, they can be explained by tensions and compressions that are transmitted along these induction lines from particle to particle, and by which the bodies are connected as if with invisible elastic springs. Likewise, Faraday sought the explanation of magnetic actions in the propagation of tensions and compressions along the magnetic force-lines, the path of which can be visualized so elegantly by means of iron filings.

How beautiful and profound that representation is becomes especially apparent when one combines it with the result of his investigation about the laws of induction currents. From the simplest form of experiments on induction by the motion of a conducting wire through a magnetic field, he was able to derive immediately the right direction for the quantitative investigation, and the clear picture of the magnetic force-lines revealed soon the law that induction phenomena are determined completely by the intersection of the force lines with the conductor.

For a moment it seemed as if the insights of Faraday would share the fate that once happened to Huygens’ major discovery, that of being suppressed by the authority of prevailing opinion. But when the profound significance of the picture that emerged for Faraday was placed in bright light by Maxwell’s investigation, these ideas, derived from one of the most elegant forms of quantitative investigation, could be combined into a great theory.

Up to now, Esteemed Audience, I have called your attention to the immediate results of quantitative research. Now, I wish to elucidate how this bears fruit for science in yet another way. In addition to these immediate results, almost every investigation contributes to the study of how one can take best advantage of the relation between different quantities, in order to measure the one by means of the other.

That study has a large influence on the development history of those measurement tools that are our most important resources for further investigations. When science in its progress replaces unsystematic observations with our senses in favor of more and more involved studies with numerical results, with which the readings of the measurement tools can be combined, then these tools become of ever growing significance.

Because of the relation that Seebeck discovered between the heating of a soldered junction of two metals and the generated current, Nobili, for example, could make use of an extremely sensitive aid for the measurement of electric current in order to determine small temperature changes of the junction. In the thermomultiplicator he obtained thereby the instrument that enabled Melloni to open a completely new field for scientific investigation.

As soon as Melloni saw this thermomultiplicator, he realized that it was the long-sought means to confirm experimentally his conviction that the sun sends us heat in invisible rays.

“*C’était un trait de lumière pour moi*,” he exclaimed {This was a flash of light for me. (Fr.)}. Henceforth, he carried this instrument with him in all his ventures, and it helped him in his struggle against hardship and ignorance, until he received the well-deserved Rumford medal from the hand of Faraday.

With the thermomultiplicator Melloni succeeded in demonstrating that the invisible rays follow the rules of the visible ones completely and differ from these only in wavelength. But the most remarkable result of the measurements with this instrument was that, by the difference in absorbance of the dark heat, the bodies must reveal in these rays a variety completely analogous to the variety that gives the visible world its blaze of colors. In the invisible world that the indication of the galvanometer reveals to us, ebonite is a translucent, and water an opaque material.

In this way, the discovery of a phenomenon that may lead us to call it the poetry of numbers, and that Melloni boldly labeled “heat coloring”, finds its origin in the development of a measuring instrument.

To name a second example, Gauss not only enriched science with a new branch, but also exerted great influence on its development by the invention of measuring tools.

Since Gauss laid the basis for the knowledge of the magnetism of the earth, the globe has been covered with stations where one follows the surprising correspondence in the capricious movements of the magnetic needle, and where, in connection with the international North-pole expedition, an important step is now taken toward explanation of magnetic storms by simultaneous observations.

These are the observations that will be performed in the far North, under the flag of the Netherlands, as a proof that in our nation the old spirit revives, that our country still supports the quest for international leadership, that there are still men for whom no sacrifice is too heavy if it is for the scientific fame of the nation, men whose fate we all follow with pounding hearts.

But beyond the significance of the knowledge of the magnetism of the earth, I call your attention to the tools invented by Gauss, because of the influence which they exerted on the development of physics in a narrower sense.

Ever since Gauss attached a small mirror to a floating magnet in order to substitute the immediate determination of the rotation by that of the shift of an image on a scale located at a considerable distance, most physicists spent their life in part behind scale and lenses. This is because the accuracy of this projection method has surpassed all expectations, and made it indispensable if the action of extremely weak forces is to be watched from some distance.

Second, to the method of measuring forces that was applied by Coulomb in the torsion-balance, Gauss added a new one by suspending a body with two wires, and succeeded in deriving from the (invariable) gravity a momentum that causes rotations in the horizontal plane.

Wilhelm Weber’s unparalleled body of electrodynamic measurements had its origin in this two-wire suspension instrument. A bifilarly suspended wire coil was used in his electrodynamometer to measure the forces that conductors of electric currents mutually exert. Thereby, he obtained the tool that is suited to study periodic and transient phenomena by means of electric currents; the tool that, after years of perseverance, enabled him to establish irrevocably the laws of the ponderomotive and induction actions, to summarize all phenomena of flowing and static electricity in a single formula, and to introduce the absolute unit system in the field of electric phenomena.

Thus we come to consider a different direction, in which quantitative investigation bears rich fruits, apart from its immediate results. Its influence on the right choice of a unit system and the establishment of measuring units is as important as its influence on the development of measuring instruments.

Similarly as with the metric system, the absolute electromagnetic-unit system of Weber was the basis for a universal language, in which henceforth all discoveries in the discipline of electricity will be written down understandably forever. The introduction of this unit system was the topic that united some weeks ago at Paris the greatest scientists in the field of electricity into one of those congresses whose modest devotion to a work of peace contrasts so encouragingly with the more publicized diplomatic efforts.

Let us dwell for a moment on the system of absolute electric units in order to examine the activities of this congress.

All measuring units for the electric quantities are derived in this system from three fundamental units, those of length, mass and time, which currently are chosen to be the centimeter, the gram, and the second. Since the gram is found in a cubic centimeter of water and the second is known everywhere from the rotation of the earth, one has only to be in possession of a standard meter in order to be able to reconstruct the whole C.G.S. system of derived units always and everywhere. In this way, for instance, the unit of force is that which gives unit acceleration to the unit of mass in one second. If one begins, in the area of electric phenomena, by considering the definition of magnetic poles as centers of forces which they exert upon each other, one can define by similar reasoning successively the electromagnetic units of the strength of the current, the galvanic resistance, and the capacitance—in short, one can measure all quantities in units that are connected to the fundamental units by strict reasoning.

The introduction of this unit system effectively ended the tremendous confusion that kept growing in the discipline of electricity in ever increasing measure, because in each investigation or instrument completely arbitrary or mutually independent units were used.

The first step in that direction was made by the British Association for the Advancement of Science with an eye on the international interests of the telegraph industry. In order to avoid at introduction {of the unit system} objections of the kind which would be raised if one had to measure in daily life lengths by means of the earth’s quadrant or the volume of the globe in liters, the British Association chose decimal multiples of the units, which corresponded best to those dimensions that were commensurate with daily practice. In naming these practical multiples the association paid a well-deserved tribute to those scientists who had contributed most to the development of the theory of electricity. The practical unit of resistance was called an ohm, that of the electric capacitance a farad. These units are suitable for material representation {in the form of standard reference materials}. The manufacturing of standard copies of the ohm and the farad was the purpose for which the British Association set out to work. The activities to realize the ohm have been particularly influential. Here one could rely upon the work of hundreds of observers: the determination of the resistance ratios had been improved to astronomical accuracies; the peculiarities of the resistance of bodies had been checked under many different conditions; the factors that influenced it had been investigated; the manufacturing of resistance standards had reached a high perfection; and Weber had performed absolute determinations of resistance with equally ingenious and accurate methods. New investigations and measurements of the British Association completed the work. When the standard wires, carefully coiled and molded into paraffin, were ready, the introduction of the absolute unit system with the ohm and the farad appeared to be guaranteed by this truly scientific endeavor, just as happened earlier with the meter and the kilogram in the case of the metric system.

At this point, considering the similarity in the development of both systems, let me recall the time when the guidance of our countryman van Swinden was requested by the National Convention at Paris for the work, which, by grand conception and energetic implementation, established triumphantly the metric system.

Having returned to our country with the fame of this achievement, the reporter of the meter commission certainly did not expect that more accurate measurements would show soon that the meter should not be considered to be a representation of the ten millionth part of the earth’s quadrant.

With the unit of the British Association things have gone as with the meter; the standard has appeared to be a pretty incorrect representation of the desired quantity. But also in another respect it shared the fate of the meter.

With respect to the latter, subsequent investigations demonstrated clearly that shape and material of the original standard were insufficient. A new congress, again meeting on invitation of the French Government, had to be appointed to give the international standard meters a definite shape. The Netherlands also contributed honorably to the results of this congress of 1872. Metrology has become a separate branch of science that represents a dry series of numbers to the outsider; but for him who appreciates the ingenious simplicity and the thoroughness of the final result, it is an inspiring and living matter. The ohm of the British Association appears now also not to supply that sufficient guarantee of invariability that is offered by the mercury-column of Simens {*sic*}. The British Association’s standard must therefore be considered as incorrect and unreliable.

With this state of affairs the French Government took again the initiative for improvement and convened a congress last year, to whose deliberations all international electric interests were entrusted but primarily the design of a general unit system. The realization which Weber’s system had found in the beautiful creation of the British Association was purified from its intrinsic defects and accepted as a permanent unit system.

The accurate establishment of the new standard ohm and farad is the important international activity, which commenced with the recently convened congress at Paris. The history of the B.A.-standard shows that the primary goal must be to perform absolute determinations of resistance by all kinds of different methods. The scientists participating in this work will be acknowledged, now that the work will be shared by several laboratories, just as it happened with astronomical problems. Only when the executive committee has received results that agree on the level of one part in a thousand, and by that approach the new sister science of metrology has advanced to a level comparable to the former, shall standards be established.

I believe I express the wish of all who are interested in the honor of our country, when I finish this review of the history of the introduction of the electric-unit system with the wish that the man who so magnificently represents the Netherlands in this area may be given the energy to participate in this outstanding international effort for a long time to come.

One has to go back to the old problems in order to appreciate fully the excellence of the absolute-unit system. This is because, in addition to certainty and unity of measurements, it provides above all clarity to the systematics of measured quantities. That clarity is rooted in the extremely simple mutual relationship that exists between the different electric and magnetic quantities and the mechanical working power, when expressed in the absolute-unit system. Thereby it elucidates clearly the induction phenomena; it predicts the melting of a wire by the discharge of a battery from the indication of the electrometer; it shows on the galvanometer the amount of horse-power that is consumed by an electric light; and it allows one to read the lift capacity of an electromagnet from the multiplicator; thereby, in short, it gives a quantitative view of the phenomena, the significance of which becomes so crystal clear for every experimenter.

Electrical measurements have contributed not only to the formation of our clear insight into electrical phenomena, which was formerly so naive. With their help the relation between light and electricity can also be unveiled.

Maxwell succeeded in calculating from the measurements of Weber and Kohlrausch the velocity with which electromagnetic oscillations in vacuum would propagate, if one assumes the existence of that weightless medium which Faraday came to consider as the carrier of the electromagnetic actions.

And see, it appeared that this velocity corresponded to that of the propagation of light, which one had already gotten to know as oscillations of a ubiquitous weightless medium. This correspondence became therefore the cornerstone of Maxwell’s theory, which sees in light nothing else than electromagnetic oscillations, a theory that has found strong support at Leiden University. And certainly in this new view of the unity of the natural phenomena it is proven most magnificently that the determination of every measurable ratio in nature adds a treasure to science.

Having outlined to you, Esteemed Audience, the role which quantitative investigation has played in establishing the general laws of nature and in providing insight into the unity of natural phenomena, I would like now to look into the near future and to ask the question whether it will retain this significance to the same degree. For this purpose I call to your attention the only new direction in the investigations of physics that stands out presently. It is the direction whose development was initiated by the discovery of the law of conservation of energy. Because of the unity of the forces of nature this law describes these as manifestations of a system of kinetic phenomena only. Now that the general laws that govern this system have become more and more known, the investigation of the structure and the combination of the parts has become prominent. One wants to gain insight into the mechanism of nature. Therefore the aim of physics is directed upon the fathoming of the nature of molecules. I would like to point out the great significance of quantitative investigation precisely in this area, for which the great problem of present-day physics is the explanation of physical properties from the structure, combination and motion of molecules.

Numerous are the difficulties associated with the solution of this problem, because the molecule itself is not accessible to us. Given the complexity of the laws that govern the connection between physical properties and the nature and motion of molecules, we may count it already an important step when we succeed in deriving approximate laws.

Their discovery is facilitated by measurements that establish the numerical values characterizing the physical properties of materials, such as density, expansion coefficient, viscosity coefficient, refractive index, conductivity etc. in different states.

Besides establishing these approximate laws the particular task of molecular physics consists in testing them with ever more accurate data.

Thus this research attains a certain correspondence with that of the course of celestial bodies.

In order to determine those, Keppler {*sic*} obtained the first approximation rule for the elliptic orbits of the planets around the sun. Newton found the basis of this rule in the general attractive force from which the course of celestial bodies could be predicted with much greater accuracy by taking into account their mutual interactions. By determining the deviations in the orbit of Uranus from those predicted by theory, Leverrier further proposed the existence of the new planet Neptunus. And if, as some have suggested, after the gravitation of Newton still another explanation principle has to be introduced, it will have to be derived from the perturbations of the most accurate measurements.

Similarly in the area of molecular physics the study of deviations from successive approximate laws is a source of new discoveries. Allow me to illustrate at this point the particular course of this research with an example.

As a first approximate law, that of Boyle-Gay-Lussac {*sic*} may be considered, according to which the product of volume and pressure of a gas is proportional to the absolute temperature. The theoretical basis of this law has been found in the consideration that the molecules of gases have no dimension and, without attracting each other, move back and forth between the walls of the space in which they are enclosed.

That this was only an approximate law, was already obvious for those gases which one could condense into a small liquid volume under a rather low pressure. The subsequent investigation of the validity of this law may be considered one of the most beautiful examples of the fertility of testing of approximate laws.

Gradually one learned to condense more gases by lowering the temperature and by raising the pressure, but a group of permanent gases still seemed to remain, to which the law of Boyle-Gay-Lussac apparently applies. Only through the investigations of Regnault (whose efficient construction and careful observance of all disturbances in each field of measurements has exerted a beneficial influence) has it been demonstrated conclusively that several of these gases correspond in their deviations from the mentioned law to {the behavior of} condensable carbonic acid {carbon dioxide}.

But the character of laws of nature becomes apparent only when one varies the measurable quantities through the entire range of possible values. The most fortunate step in the further investigation of these deviations was that of Andrews. He subjected the carbonic acid inside capillary glass tubes at different temperatures to very high pressures. Thereby, a material like carbonic acid appeared to deviate so much from the rule of Boyle-Gay-Lussac, that a continuous transition between the gaseous and the liquid state could even be demonstrated, and Andrews found in the critical temperature the limit below which a gas has to be cooled in order to be condensed into liquid. The significance of the critical temperature was further demonstrated magnificently when the permanent gases yielded to the genius of Cailletet and Pictet, who succeeded in observing oxygen and hydrogen as a mist or jet of liquid at extremely low temperatures.

From the measurements of Andrews the graphical representation of the law connecting pressure, volume and temperature was derived from nature. In a similarly accurate and complete way natural phenomena have to be described before they can be fathomed. By his ingenious investigations, it was given to our countryman Van der Waals to shed the full light of theory on the deviations from Boyle’s law and on the continuity of the gaseous and the liquid state, and to explain this from the motion of molecules. (In that context he had to pay attention to the volume of the molecules themselves and to the attraction that they mutually exert, which could be accounted for as molecular pressure). By his theoretical result (that the product of the free volume and the total pressure, which keeps the collisions in equilibrium, must be proportional to the absolute temperature) he stimulated numerous measurements in this field. He could even predict the critical temperature of a gas from its deviations from the rule of Boyle-Gay-Lussac.

The explanation of the critical state was only the first step taken by van der Waals to develop a molecular theory of liquids. Soon he discovered that the critical state provides the means to express the properties of liquids in corresponding states, which are of highest importance for intercomparisons. One transfers different liquids into these corresponding states, when, at temperatures that have the same proportion to the critical temperatures, one subjects them to pressures that are in the same proportion to the critical pressures. In these corresponding states, the volumes that are occupied by the same number of molecules, are then for every liquid the same part of the critical volume. Using the wealth of measurements which Regnault, Kopp, and others had collected about the physical constants of liquids, this new approximation rule appeared to be of broad validity. By adding a surprising new perspective, this concept enlivens their numerous investigations (about expansion coefficients, compressibility coefficients, vapor pressures, and latent heat).

The discovery of these corresponding states raises in turn the question of the basis of their remarkable significance.

It seems to me, that the analogous behavior of liquids in these states finds its explanation in the correspondence of the collective motion of the molecules, which one names the similarity of motions. [Considering liquids as systems of hard, elastic, similar molecules, which influence each other with forces inversely proportional to a power of the distance, then the motions of these molecule systems in corresponding states may be viewed as similar motions. To exemplify this similarity we may point to the correspondence of tools machined from the same design drawing but on different scales and from different materials. To complete the picture we have then to suppose that the one tool runs faster, the other slower, all according to dimensions and material. Such differences and correspondences as exist between different tools appear to exist between the molecular systems we call liquids, when they are put in corresponding states. Suppose that we derive from each molecular system the measures of mass, length and time with which the states of this system can be described in the simplest way. Nature provides such units for instance in the mass of the molecule, in the length of the radius of the (spherical) molecule, and in the time during which two molecules at unit distance would give each other unit acceleration. Upon these units, which are independent of our arbitrariness, we can build a system of derived measuring-units (of pressure, living force {energy}, temperature etc.) in a similar manner as with the absolute electromagnetic measuring system. We might give it the name of molecular absolute measuring system. Corresponding motion-states of those molecular systems that we call liquids are now those that are expressed by the same numbers when each is described in its own system of units.] Consequently one can derive from the behavior of a single liquid that of every other by means of some proportionality factors.

Just as with every other approximate law, so also the rule of van der Waals provided a new stimulus for measuring investigations. I even dare to claim that his discovery of the corresponding states causes a complete reversal in the field of the determination of the physical constants. Instead of the arbitrary scale of temperature and pressure, his discovery points to the natural scale according to which the physical properties of liquids can be stated comparably. Specific heat; the coefficients of friction, of heat conductivity, and of capillarity; dielectric polarization; molecular refractivity; and dispersion have to be studied according to this scale of corresponding states. Only then may one expect to find approximate laws of more general validity than those which were deduced up to now, approximate laws with the characteristic that every deviation from them gives information for the inquiry into the nature of the molecules. This is because in those deviations the characteristic nature of structure and interactions of molecules will display itself precisely.

As in every part of molecular physics, theory and measurement must join hands here in any further investigations. From approximated conjectures about molecules, theory can provide valuable guidance for experiments. In turn, measurement decides about their correctness; it makes sure that research is not lulled into a false sense of certainty by the enticing agreement of rounded data with an elaborate theory. Rather, in the deviations it provides the natural building materials for new hypotheses about the properties of the molecules.

The extension of the limits of investigation (concerning temperature and pressure), and the increase of accuracy have to keep pace with the continuing reduction of the deviations to ever more refined approximate laws. Thereby the pumps of Cailletet and Pictet become on the one hand indispensable laboratory instruments; on the other hand a physical laboratory must be organized in part like an astronomical observatory. It must be provided with instruments whose characteristics are wholly known and recorded in registers, and of rooms suited to use these instruments with success.

Having talked about the new direction of physics, a few comments about the new connection of physics and chemistry are in order. More and more they merge into one great science, the mechanics of atoms. My goal, to promote the convergence of these two fields through the work of this physical laboratory, is explained best by considering for a moment their numerous mutual interests in measurement investigation. The relation between vapor density and molecular weight, the significance of the valence in electrolysis, and the influence of physical considerations on the choice of comparable atomic weights, whose arrangement in Lothar Meyer’s table conveys to us one of the most important laws of nature, are some topics of mutual interest. The boundaries between physical and chemical effects may be considered simply superficial. Both the dynamic approach to chemistry, which sees everywhere various reactions competing with each other in chemical processes (until their equilibrium has been reached according to the second law of mechanical heat theory), as well as the static approach, which tries to reveal the molecular structures in the most detailed manner by means of the periodic table of elements, require measurement investigations for their development. The dynamic approach has to study dissociation, both by deviations from Avogadro’s law as well as by the deviations of the properties of liquids in corresponding states. It has to follow the paths of reactions (applying electricity, according to Faraday a rich source of insight) by absolute measurements of the electromotive force, with which Bosscha initiated the mechanical theory of electrolysis (as well as that of the velocity of electrolytic motion). From Berthelot’s and Thomsen’s {*sic*} numerous measurements (about the difference in energy at the beginning and the end of chemical conversions, combined with the most ingenious choice of the reactions) the dynamic method sheds light on the big problem, whether or not chemical reactions will take place or will be reversible, and it gives us a foretaste of its development, when it will be freed from the ties by which it is still bound due to the low state of development of molecular physics.

Concerning the static approach, it relies more and more on knowledge of physical properties to determine the characteristics of the structure of molecules. Determinations of the heat of formation of isomeric materials (Berthelot’s kenomeries {systematic investigations of the structure-property relationship}), of boiling points and vapor pressures (represented by curved lines), of specific volumes and viscosity coefficients (Lothar Meyer), all have to serve to collect building blocks in this direction. The investigations of Landolt and Brühl have introduced the spectrometer for the tracing of multiple bonds in chemistry, but it seems to me that the most characteristic differences in the structure of the molecules will be discovered by the investigation of the thermal properties (heat conductivity, viscosity coefficients, etc.) in corresponding states. However, just as the measurements of Picard and Flamsteed alone enabled Newton to deduce the rule of the general attraction force, so is it also necessary to compile tables of the physical properties of all sorts of materials in various states by measurement and theory, tables like those of the orbits of the celestial bodies, before the synthesis of indigo can be carried out in a physical experiment, the investigations of Regnault will be considered chemical measurements, and a second Newton will explain the world of molecules.

Undoubtedly, future scientific advances depend on quantitative investigation. Likewise, a place of honor in the laboratory belongs to replications of investigations that have been performed with classical accuracy because I consider these as the best school for the education of physicists in the true sense of the word.

Repetition of an investigation can stir interest only when it happens in a form that has been identified by the researcher as the most suitable. My feeling is, however, that the mere observation of a phenomenon according to certain protocols is somewhat unsatisfactory. But a quantitative investigation includes the check whereby one can satisfactorily see the fruits of one’s own labor in the accuracy of the result.

Above all it requires a clear understanding of those quantities one wants to measure. The goal of reaching the ultimate accuracy with appropriate means teaches one to examine their suitability and forces the researcher to exercise care and perseverance. Results that give self-confidence can be obtained only by self-criticism and method.

Through the completeness with which each observation has to be examined in quantitative investigations, the gift to observe new phenomena is developed in many an eye.

Investigations should excel by innovation as much as by solidity. And that solidity is cultivated when one studies the classics as is necessary to repeat their measurements successfully. Indeed, physics does have classics, the study of which is the ever fresh source of new insight and inspiration for scientific work.

They teach that one has to examine the phenomena up to a limit that corresponds to the general state of science. A multitude of essays in the present scientific literature give the impression that the author aims to connect his name to as many phenomena as possible. In contrast, I wish to develop a scientific ethic, under the guidance of the classics, that demands complete and thorough research.

Dear Sirs Curators!

I thank you most cordially for the confidence, which you have placed in me, now that you have appointed me to this highly important task, notwithstanding my youthful age, and found my preparations for that purpose to be fruitful.

In establishing two professorships for physics at this university, your broad view has already acknowledged the importance of a complete devotion to the rich treasures that are stored in the cabinet of physics. I promise to make the most of these treasures to the best of my knowledge.

It would certainly be desirable to have at least one “world-class” physics laboratory in the Netherlands. One individual citizen of Baltimore founded the magnificent John Hopkins-University {*sic*}. The Duke of Devonshire presented to Cambridge the splendidly equipped laboratory, which boasts the name of Cavendish. I consider it to be no idle hope that our country, with the revival of national spirit, will be able to point to men like a John Hopkins and a Duke of Devonshire. Then it will be possible to name one of the re-created laboratories of our universities after Huygens, as a long-indebted homage of the nation that counts this peer of Newton among her greatest sons. As long as the scientific spirit of our country has not risen to this level, I will need your support in the fullest measure to work fruitfully at Leiden University along the direction that I have tried to outline to you. I hope you will not deny me that support.

I would have appreciated very much to see my highly esteemed predecessor Rijke here in order to express to him what a difficult task it is for me to become his successor. I will have to give all my energy not to be inferior to him in the zeal with which he has always devoted himself to his carefully prepared courses, and in experimental skill, which he acquired in a lifetime of devotion to experimental physics.

It is only by law that he has to retire from that field of work where his activity still could have produced the finest fruits.

It may be considered a task for one of his numerous students to dedicate a word of gratitude for his contributions to scientific education; for me it is a pleasant duty to praise here the excellent care which has been paid by him to the foundation of the cabinet {laboratory} of physics, and to express that he is for me a brilliant example to follow in judiciously expanding and carefully managing this valuable collection {of instruments}.

May it be given to him for many years to enrich science with contributions, which are not for me to praise when a Tyndall says that their merit matches the modesty of the discoverer.

Dear Sirs Professors!

Entering the circle of men with so many achievements in science and education, I ask you with confidence to support me with your experience and your benevolence.

That it will be a pleasant task for me to cooperate particularly with you, highly esteemed colleagues of the Faculty of Mathematics and Physics, I have sensed already from the politeness with which many of you have received me. If I may mention one experience of life, then it is this, that by unselfish devotion to common ideals the firmest ties of friendship are formed. Before my appointment, highly esteemed Van De Sande Bakhuyzen, I could already experience from you the most pleasant signs of affection. You, highly esteemed Van Bemmelen have welcomed me, now as a colleague, with no less heart-felt friendship than that with which you earlier awakened in the youthful student the desire for science, and with which you have always shown him the most cordial interest. I share this privilege with you, highly esteemed Lorentz. In your lasting friendship I see the guarantee that at Leiden University that close connection of theory and experiment will be fostered which the new direction in our science rightly demands.

Highly Esteemed Bosscha!

I do not need many words to tell you with what deep gratitude I am addressing you from this place. In the four years during which I was your student as assistant, you prepared me for and assigned me to activities of ever more responsibility. Your good expectations are to me a strong support in the beginning of the task, in the accomplishment of which I hope to continue the privilege of considering myself as your student. It will be difficult for me to become for others what you have been for me. It will be my highest goal to show that you did not grant me your precious time in vain when you tried to communicate to me your insights, your clarity of presentation, your talent to reduce scientific problems to their simplest shape, and to show that I was not unworthy to pass through a school of experimental research under your invaluable guidance. Continue to grant me, besides your support and your information, that friendship which I consider to be one of the richest treasures of my life.

Dear Sirs Students!

The university must be the place where striving at ideals is nurtured. The *spes patriae* {hope of the fatherland (Lat.)} is entrusted to her not only to be equipped with that knowledge which society needs in order to exist and to function, but primarily to spread among the people the inspiration for noble motives, which is the indispensable basis of a strong national identity. Do not deny me your cooperation, when I try to contribute my part to the cultivation of an understanding of the scientific method instead of just reporting results, in order to make the advancement of science a goal for all people.

I Have Spoken.

## 4. Epilogue

Kamerlingh Onnes’ speech is a unique document that reverberates to this day with those who are engaged in metrology and in research on the thermophysical properties of fluids. In fact, the Nobel Prize for Kamerlingh Onnes cited “…his investigations on the **properties of matter** at **low temperatures** {emphases added} which led, *inter alia*, to the production of liquid helium” as his primary achievements. The following remarks offer some thoughts about the current state of affairs in these fields taking selected aspects of the work of Kamerlingh Onnes as a reference. The full breadth of Kamerlingh Onnes’ achievements has been cited and appreciated elsewhere.

Kamerlingh Onnes’ main quest was the condensation of helium, the last gas for which the gas-to-liquid phase transition remained to be demonstrated. He achieved this goal in 1908, 26 years after his inaugural speech, through painstaking developments of the necessary instrumentation to conduct experiments at ever lower temperatures. Eventually, he reached a lowest temperature of less than 0.9 K. However, his endeavor was much broader than building just a single instrument. The laboratory he built at Leiden can be regarded as a precursor of a national metrology laboratory.

Another form of condensation was achieved in recent years. Cooling some 2000 rubidium atoms to 170 nanokelvins, Anderson et al. observed in 1995 for the first time a Bose-Einstein condensate [17]. In 2001, this group of scientists observed the dynamics of Bose-Einstein condensates at temperatures around three nanokelvins [18], and two of this group were honored with the Nobel Prize in physics, following in the very footsteps of Kamerlingh Onnes. It is noteworthy from an organisational viewpoint that this advance was accomplished in a research partnership with a national metrology laboratory, i.e., the National Institute of Standards and Technology (NIST). It is noteworthy from a scientific viewpoint that the achieved temperature had been lowered by eight orders of magnitude in the nine decades since Kamerlingh Onnes had condensed helium. It is helpful to consider this advance in the following perspective. The estimated temperature at the surface of the sun is close to 6000 kelvins, just slightly over one order of magnitude greater than the ambient temperature on earth, if this is taken as 300 K. A temperature of 3 nK is 11 orders of magnitude lower than ambient temperature, surpassing the temperature ratio between the surface of the sun and the ambient on earth by almost ten orders of magnitude.

In his lecture of 1959, Feynman called the field of low-temperature research “seemingly bottomless” [1]. It is well justified to delete the attribute “seemingly” because the Third Law of Thermodynamics, enunciated by Nernst in 1906 [19], implies the unattainability of absolute zero. In other words, absolute zero is a singularity and one can indeed go infinitely down and down in temperature without ever reaching it. The international temperature scales reflect absolute zero as a “point” rather than as a singularity because they are based at low temperatures on the ideal gas law, which is indifferent to the meaning of temperature [20]. The indifference is removed when corrections are added to account for the behavior of real gases. Kamerlingh Onnes wrote in 1901 an equation of state in polynomial form

$$pv=RT\left(1+\frac{B\left(T\right)}{v}+\frac{C\left(T\right)}{v^{2}}+\ldots \right)$$ (1)

and suggested to call *B*(*T*), *C*(*T*), … “virial coefficients.” Here, *p* denotes pressure, *v* the molar volume, *T* the absolute temperature, and *R* the universal gas constant. Mason and Spurling noted the special importance of the virial equation of state because of its sound theoretical foundation [21]. One approach is to calculate the pressure from one of the partition functions of statistical mechanics. This introduces the inverse temperature *β* = 1/*T* as the natural variable that reflects the unattainability of absolute zero, and which as early as 1964 was called “cryos” by a countryman of Kamerlingh Onnes, Professor Frederik Belinfante, then of Purdue University [22].

The appropriateness of an inverse temperature scale in view of the Third Law of Thermodynamics has been mentioned by Zemansky [23] and others even though such propositions are often based on considerations of the opposite extreme, the infinite-temperature limit. The rapid progress of low-temperature physics in going ever further down will create a growing need for a measure that is appropriate in that domain and thermodynamically consistent with the divergence at absolute zero. The present empirical temperature scale does not need to be replaced by an inverse temperature scale because it is consistent with the physiological sensation of hot and cold and thus useful. Rather, the linear scale and the more logical *β*-scale should be embraced as complementary measures similar to the complementary use of reciprocal quantities such as density and volume, viscosity and fluidity, or conductance and resistance. It is noteworthy that our understanding of temperature has been expanded recently by developments of statistical thermodynamics and computer simulation [24, 25].

Kamerlingh Onnes’ speech was full of enthusiasm about the prospects that the development of experimental science into metrology offered. It culminated in the famous motto “Through measurement to knowledge.” However, he had a balanced view of the mutual fertilization between experiment and theory, and gave credit to van der Waals, whose work “stimulated numerous measurements in this field.”

**Fig. 1:**
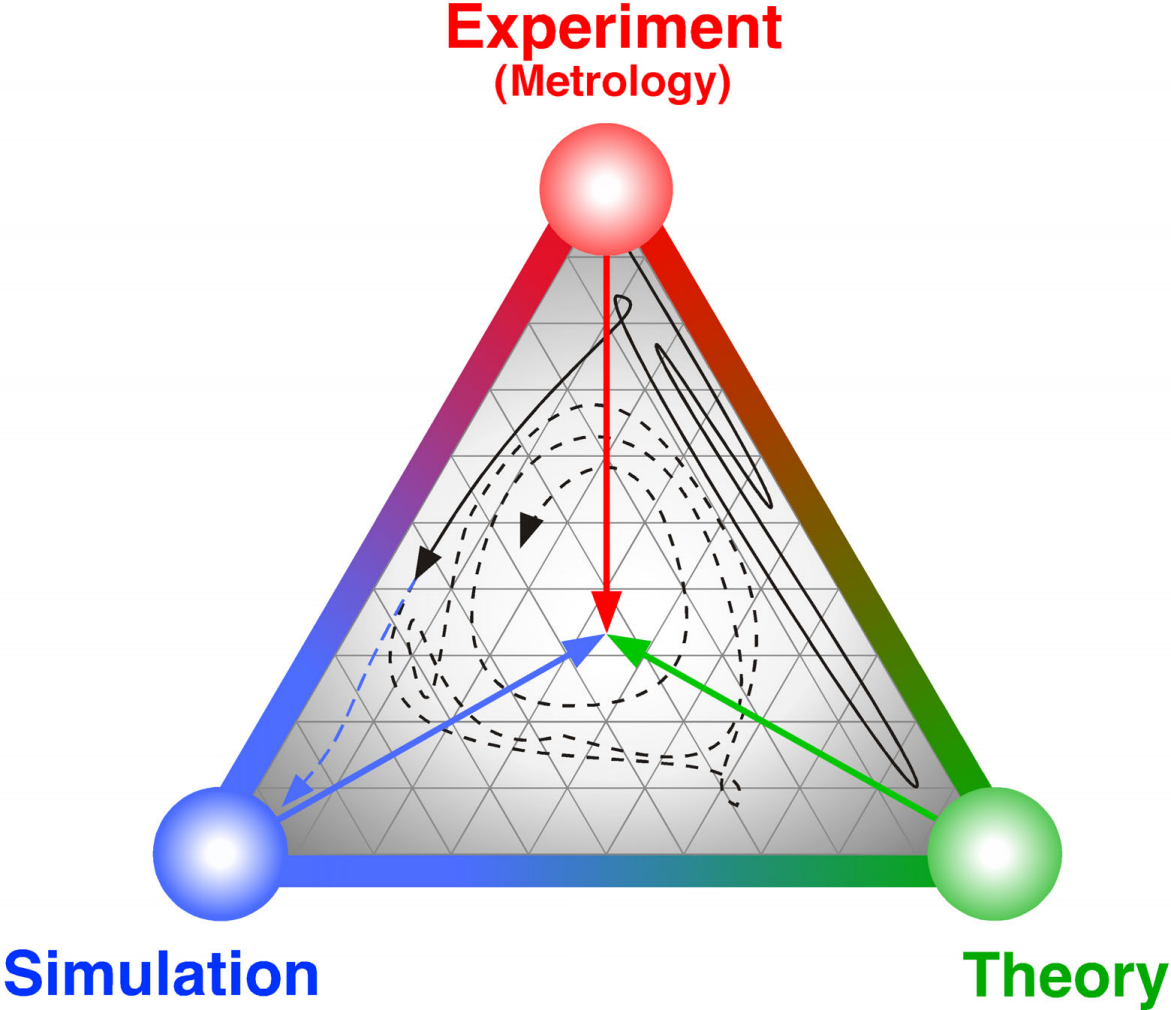
The complementarity of experiment, theory, and simulation may be illustrated by a phase diagram of a ternary system. The balance between the three components lies in the center of the triangle. The full black line represents the early binary interaction between experiment and theory and the rise of simulation. Its end point approximates the present state of affairs. The blue dashed line indicates the “Experiments—no thank you!” scenario [30]. The black dashed line suggests a more probable future development. The system may have other dimensions not yet known as computer simulation was not perceived when Kamerlingh Onnes gave his inaugural lecture in 1882.

This binary system of knowledge acquisition through the interplay of experiment and theory has expanded into a ternary system with the advent of computers and the development of methods to employ them. Fig. 1 is an attempt to illustrate this interplay using an analog from mixture thermodynamics. Computer simulation of thermodynamic fluid properties began with the Metropolis algorithm [26, 27], which forms the basis of Monte Carlo simulations by applied statistical mechanics. The molecular-dynamics technique originated a short time later in the work of Alder and Wainwright [28, 29]. This expanded simulation capabilities to transport properties. Propelled by an exponential doubling of processor speed every 18 months (“Moore’s law”), computer simulation is now becoming a third method of knowledge acquisition that complements experiment and theory. Some protagonists seem to be overwhelmed by enthusiasm and suggest a superiority of computer simulations over measurements [30]. This attitude reminds one of 1 Cor. 12:21: “The eye cannot say to the hand, ‘I have no need of you,’ nor can the head say to the feet, ‘I have no need of you.’ ” [31]. There are at least two reasons why measurement will not be made obsolete by computer simulation. One is of a cognitive nature and the other is of an organizational nature. Kamerlingh Onnes’ speech provides guidance in both respects.

The cognitive difference between measurements and simulations can be stated quite simply, as follows. Simulations observe the behavior of a model system in a given thermodynamic state under atomic and/or molecular interaction rules (electron wave functions and/or force fields), which are given by the programmer to approximate the real interactions. Measurements, on the other hand, aim to confine a sample as well as possible into a thermodynamic state, but the interaction rules are not given. Rather, they are inferred from the observed macroscopic response of the sample. The difference is obvious. “There is clearly more potential for surprise and discovery in measurements.” [32].

The organizational difference appears when the framework of standards into which metrology has matured [33] is compared with the development of computer simulation over the last five decades. Uncertainty and precision are mandatory attributes of every experimental result, while “…making uncertainties evident is a tough challenge for computer simulations [34].” It is a logical task for national metrology laboratories to be involved in the organization of computer simulation by becoming clearinghouses for simulation methods and results, by developing protocols, and by setting benchmarks. NIST has taken a lead by co-organizing the First Industrial Fluid Properties Simulation Challenge that will conclude in September 2002 [35].

The greatest benefit will be derived when all three methods of knowledge acquisition are employed to complement each other. Kamerlingh Onnes said in his inaugural speech 1882 “The character of laws of nature becomes apparent only when one varies measurable quantities through the entire range of possible values.” This was reiterated by Quinn, in 1994: “Physical theory is reliable only to the extent that its predictions can be verified quantitatively…” [36, 37]. One example where computer simulation has advanced metrology quantitatively is the *ab initio* calculation of thermophysical properties of ^3^He, ^4^He, and their mixtures with smaller uncertainties than have been achieved in measurements [38, 39]. The simulation results provide improved calibration standards for instruments relying on these thermophysical properties. This mutual fertilization will be promoted further by developments such as the recent expansion of the publication scope of a journal that used to communicate experimental data exclusively [40]. In such a joint forum, simulators will have to familiarize themselves more with experimental protocols, methods, and results, while experimenters will have to concentrate on measurements that cannot be carried out at comparable accuracy by computer simulation. Connecting computer simulations with physical reality is an emerging area for standardization [41, 42], to which national metrology laboratories can contribute their unsurpassed competence in measurements.

The exponentially accelerating capabilities of computer simulation overshadow presently a nascent breakthrough of similar magnitude in experimental science. What Feynman envisioned in his historic lecture [1], namely “to go down and down” in scale, is now becoming reality. Miniaturization gives access to domains of states of matter that were unthinkable only a decade ago. Besides, miniaturized experiments can be carried out faster and therefore can be run in parallel, which makes combinatorial methods possible. This parallel mode of operation will not only make it possible to measure one thermophysical property of a binary or multicomponent system at several compositions at once. It will be also possible to connect micromachined sensors for several properties (density, heat capacity, viscosity, dielectric constant etc.) in series for additional gains in speed of measurements [43]. These developments will change experimental science profoundly and will lead to a rediscovery of measurements as a method to probe molecular interactions. Most likely, they would move a leader of the foresight of Kamerlingh Onnes to give the same enthusiastic speech about the prospects of metrology today as he did in 1882.

In his opening essay for the “Pathways of Discovery” series in Science magazine during the year 2000, Stephen Jay Gould [44] wrote that “…scientists should cherish good historical analysis because real, gutsy, flawed, socially embedded history of science is so immeasurably more interesting and accurate than the usual cardboard pap about marches to truth fueled by reason and observation (‘the scientific method’). … Most daily activity in science can only be described as tedious and boring, not to mention expensive and frustrating. Thomas Edison was just about right in his famous formula for invention as 1 % inspiration mixed with 99 % perspiration. How could scientists ever muster the energy and stamina to clean cages, run gels, calibrate instruments, and replicate experiments, if they did not believe that such exacting, mindless, and repetitious activities can reveal truthful information about a real world?” Kamerlingh Onnes’ work is an example for such perspiration, to which we have added his aspiration by translating his inaugural speech of 1882. It appeared fitting to do this on the occasion of the Centennial of the National Institute of Standards and Technology because nowhere else is perspiration as important as in the work of standards laboratories.

## Footnotes

1 “Thought is only a flash between two long nights, but this flash is everything.” Jules Henri Poincaré, French mathematician (1854–1912), a contemporary of Heike Kamerlingh Onnes.

2 Dutch words such as “physics” and “truth” were of feminine gender in 1882 while in modern Dutch and in English they are not. Here, the feminine gender was retained to convey to the reader the flavor of the original.

3 This is a more literal translation of the original Dutch text, which uses the form of gerunds. Such construction is not common in English. The translation of Mendelssohn [9] with nouns is preferable, as in the title of this paper: Through Measurement to Knowledge.
